# Relationship between emotional labor strategies and the quality of work life of operating room nurses

**DOI:** 10.3389/fpubh.2024.1503605

**Published:** 2025-01-09

**Authors:** Li Li, Yingjuan Cao, Yinuo Sun, Yue Li, Yonghua Zhai, Yan Yan

**Affiliations:** ^1^Department of Operating Room, Qilu Hospital, Shandong University, Jinan, Shandong, China; ^2^Department of Nursing, Qilu Hospital, Cheeloo College of Medicine, Shandong University, Jinan, Shandong, China; ^3^School of Nursing and Rehabilitation, Shandong University, Jinan, Shandong, China; ^4^Nursing Theory and Practice Innovation Research Center, Shandong University, Jinan, Shandong, China

**Keywords:** emotional labor, Work-Related Quality of Life, nurse, quality of work life, operating room

## Abstract

**Introduction:**

Emotional labor involves regulating one’s emotional state at work to align with organizational expectations. For operating room (OR) nurses, emotional labor is an inherent part of their roles, with different strategies potentially impacting their work-related quality of life (WRQoL) in distinct ways. This study aimed to examine the relationship between emotional labor strategies and WRQoL among OR nurses.

**Methods:**

A cross-sectional study was conducted using convenience sampling, recruiting 395 OR nurses from 11 secondary and tertiary hospitals in Shandong Province. Data were collected using the General Information Questionnaire, Emotional Labor Scale, and Work-Related Quality of Life scale. Statistical analyses, including descriptive statistics, Pearson correlation analysis, and multiple stratified regression, were applied to explore the relationships between variables.

**Results:**

A total of 372 valid responses were obtained, with an effective response rate of 94.18%. The mean WRQoL score was 114.17 (*SD* = 16.54). Among emotional labor strategies, expressing naturally felt emotions was the most frequently used. Surface acting showed a significant negative correlation with WRQoL (*r* = −0.437, *p* < 0.05), while deep acting and expressing naturally felt emotions were positively correlated with WRQoL (*r* = 0.291 and *r* = 0.457, respectively, *p* < 0.05). Multiple stratified regression analysis confirmed that emotional labor strategies significantly influenced WRQoL.

**Conclusion:**

Emotional labor strategies play a crucial role in shaping the WRQoL of OR nurses. Nursing managers should prioritize assessing nurses’ emotional labor status and provide targeted guidance to promote positive strategies, such as deep acting and expressing naturally felt emotions. Such measures can enhance nurses’ physical and mental well-being, ultimately improving their quality of work life.

## 1 Introduction

In April 2022, the National Health Commission of China issued the “National Nursing Development Plan (2021–2025),” designating “strengthening the construction of the nursing team” as one of the primary tasks (1). As the reform of the medical and healthcare system deepens and the scientific level of nursing management continues to improve, the issue of how to effectively mobilize and stimulate the enthusiasm of nurses, while simultaneously creating a supportive and empowering professional environment, has become an increasingly important topic for healthcare institutions. Ensuring that nurses are motivated and satisfied with their work environment is essential not only for their own well-being but also for improving the quality of healthcare services provided to patients.

Among the various factors that contribute to a positive professional environment, the quality of work life (QWL) has emerged as a critical indicator for evaluating the overall work environment of nurses. QWL refers to the extent to which nurses’ personal needs are met while also fulfilling organizational goals and objectives (2). A substantial body of research has shown that QWL is closely related to both the well-being of individual nurses and the overall performance of the organization. A high QWL is associated with greater job satisfaction, reduced burnout, lower turnover rates, and improved performance (3, 4). On the other hand, a low QWL is closely linked to job burnout, high turnover intentions, and reduced job satisfaction, which can lead to a higher attrition rate and, ultimately, a decrease in the overall quality of healthcare services provided (3, 4). Therefore, prioritizing QWL is crucial for enhancing nurse retention, improving patient care, and fostering a more efficient healthcare system.

Research on QWL among healthcare workers has identified several factors that influence this aspect of nursing, such as the quality of organizational management, interpersonal relationships with colleagues, family income, workload, and individual personality traits (5–7). Additionally, emotional labor has been recognized as one of the most significant factors affecting nurses’ quality of work life (8, 9). Emotional labor refers to the process of regulating one’s emotional state at work to meet organizational expectations and requirements (10). In the operating room (OR), emotional labor is particularly prevalent, as OR nurses must constantly adjust their emotional states to collaborate with surgeons, anesthesiologists, and other healthcare professionals, communicate effectively with patients and their families, and respond to unexpected situations such as patient emergencies or equipment malfunctions. The emotional demands placed on OR nurses can be particularly intense, making emotional labor a key element of their daily work experience. Emotional labor has been conceptualized as a three-dimensional construct, which includes surface acting, deep acting, and the expression of naturally felt emotions (11, 12). Surface acting involves the outward display of emotions that are not genuinely felt, often requiring nurses to suppress or hide their true feelings to meet workplace emotional expectations. Deep acting, on the other hand, involves a more profound internal change, where the nurse alters their emotional state to align with the required emotional display. The expression of naturally felt emotions occurs when nurses experience and express emotions that are congruent with the emotional situation at hand. While these three strategies have been identified in general, the specific emotional labor strategies adopted by OR nurses remain unclear and under-researched (11).

According to the Job Demands-Resources (JD-R) model (13), the variables of emotional labor strategies, workload, and quality of work life investigated in this study can be understood through the framework of this model. The JD-R model posits that both job demands and resources are critical factors influencing employee performance and well-being. In this study, emotional labor strategies are considered “job resources” because effective emotional labor strategies help employees cope with work pressures, enhance job performance, and improve overall well-being. Strategies such as deep acting and surface acting are ways employees regulate their emotional states in response to emotional demands. According to the JD-R model, when employees have access to sufficient emotional resources (e.g., appropriate emotional labor strategies), they are more likely to manage job demands effectively, which in turn positively impacts their QWL. On the other hand, workload is categorized as a “job demand” in the JD-R model, as it represents the pressure and stress that employees face. High workloads, especially for operating room nurses, are a significant source of strain, potentially leading to burnout and lower QWL. The JD-R model suggests that when job demands exceed available resources, it can negatively affect employees’ job satisfaction, well-being, and overall work-life quality.

To the best of our knowledge, there is limited research on the current state of emotional labor and QWL among OR nurses, and the influence of emotional labor strategies on QWL has yet to be comprehensively explored. This study aims to bridge this gap by investigating the current status of emotional labor strategies and QWL among OR nurses. Additionally, it will examine how demographic factors, such as age, gender, years of experience, and job roles, along with different emotional labor strategies, affect nurses’ quality of work life. The findings from this study are expected to provide valuable insights for nursing managers, helping them develop effective management strategies, offer appropriate organizational support, and guide nurses in adopting emotional labor strategies that enhance their well-being. Ultimately, the study aims to improve the quality of work life for OR nurses, which in turn will contribute to a higher quality of patient care and a more efficient healthcare environment.

## 2 Materials and methods

### 2.1 Study design and sample

This study was a cross-sectional survey conducted between September and October 2022. The study participants were OR nurses from 11 secondary hospitals or higher in Shandong Province, China, selected through convenience sampling. The inclusion criteria for the study were: (1) holding a valid nurse practicing qualification certificate, (2) working for at least 1 year, (3) providing informed consent, and (4) voluntary participation in the study. The exclusion criteria were: (1) nurses who had been on maternity or sick leave for more than 3 months, (2) interns, and (3) nursing students.

For sample size estimation, the number of participants was calculated based on the guideline of 5–10 participants per scale item (14). Given that the questionnaire in this study included a maximum of 33 items, the estimated sample size ranged from 165 to 330 participants. Considering a 10% invalid response rate, the final required sample size was 182–363 participants. In this study, a total of 372 valid responses were collected, ensuring sufficient statistical power for the analysis.

### 2.2 Measurements

#### 2.2.1 Demographic details

To gather demographic information, we utilized the “General Information Questionnaire,” which was developed by our research team. The questionnaire assessed several key factors, including sex, age, hospital level, interpersonal relationships, educational background, professional title, years of employment, job position, marital status, number of children, average income, and average working hours.

#### 2.2.2 Emotional labor strategies

The Emotional Labor Scale (ELS), developed by Diefendorff et al. and later translated by Bai, was used to assess the emotional labor expression strategies in this study (11, 15). This scale consists of 14 items across three dimensions: surface acting (7 items), deep acting (4 items), and the expression of naturally felt emotions (3 items). The Cronbach’s *α* coefficients for these dimensions were 0.750, 0.721, and 0.718, respectively, indicating satisfactory internal consistency. The scale uses a Likert 5-point scale, with higher scores indicating a more frequent use of the corresponding emotional labor strategy. Previous studies on the use of this scale among nurses have demonstrated its good reliability and validity.

#### 2.2.3 Quality of work life

The “Work-Related Quality of Life Scale-2 (WRQoL-2)” developed by Van-Laar et al. and translated and revised by Shao et al., was used to assess the quality of work life among OR nurses (16, 17). The scale comprises 33 items across seven dimensions: working conditions, work pressure, work control, work-family balance, job evaluation, general happiness, and career satisfaction. Additionally, there is one independent item for the overall evaluation of personal work-life quality. The Chinese version of the WRQoL-2 has a Cronbach’s *α* coefficient of 0.939, with individual dimensions ranging from 0.652 to 0.859. The scale uses a Likert 5-point scale, where higher scores indicate better work-life quality, with reverse-scored items appropriately adjusted.

### 2.3 Investigation methods and quality control

To ensure the smooth progress of the study, the research team first contacted the directors of the operating rooms at each hospital, who then arranged for the relevant personnel to assist in coordinating the study. Prior to the survey, the researchers provided detailed training for the hospital heads, covering the content of the questionnaire, data collection methods, and quality control procedures. The questionnaires were distributed via the “Questionnaire Star” platform, with survey sessions scheduled during departmental meetings or morning meetings to ensure timely completion. The first page of the questionnaire included standardized instructions, outlining the purpose of the survey, its scientific research use, and the response guidelines. All questionnaires were completed anonymously, and each IP address was allowed only one submission. All items in the questionnaire were mandatory to ensure the completeness of the data. After the questionnaires were collected, the research team conducted a strict quality audit, excluding any questionnaires that did not follow basic logical consistency or showed obvious patterns of error, thereby ensuring the accuracy and reliability of the data.

### 2.4 Statistical methods

Data analysis was performed using SPSS 20.0 software. For continuous variables, those with a normal distribution were described using mean and standard deviation; for non-normally distributed data, median and interquartile range were used. For categorical variables, frequency and percentage were used for description. Group comparisons for continuous variables were conducted using independent t-tests or Mann–Whitney U tests, and for categorical variables, chi-square tests or Fisher’s exact tests were used. Pearson correlation analysis was applied to evaluate the linear relationship between emotional labor strategies and WRQoL. To further explore the predictive role of emotional labor strategies on WRQoL, multiple linear regression analysis was used. All statistical analyses were two-tailed, with a *p*-value of <0.05 indicating statistical significance.

## 3 Results

### 3.1 General demographic information

Table 1 illustrates the demographic characteristics of the 372 OR nurses. The majority were female (86.6%, 322/372), predominantly aged between 31 and 40 years (48.9%, 182/372), with 11–20 years of working experience (33.3%, 124/372). Most participants held a bachelor’s degree (84.7%, 315/372) and were married (82.0%, 305/372).

**Table 1:** Univariate analysis of QWL of OR nurses with different characteristics (*n* = 372).

Subjects	Frequency	Percentage (%)	Score of QWL (χ ± s)	*T* or *F* value	*p* value
Sex					
Male	50	13.4	110.28 ± 17.95	−1.793	0.074
Female	322	86.6	114.77 ± 16.25		
Age (years)					
≤ 25	29	7.8	113.24 ± 15.87	0.521	0.668
26–30	77	20.7	113.90 ± 16.26		
31–40	182	48.9	113.53 ± 17.58		
>40	84	22.6	116.13 ± 14.70		
Hospital-grade					
Third Grade First Class Hospital	215	57.8	114.59 ± 16.04	0.181	0.834
Third Grade Second Class Hospital	63	16.9	113.89 ± 19.06		
Secondary Grade Hospital	94	25.3	113.39 ± 16.00		
Employment way					
Contractual system	187	50.3	113.89 ± 17.12	0.376	0.687
Personnel agency system	44	11.8	112.70 ± 14.79		
Formal establishment	141	37.9	115.00 ± 16.32		
Education level					
Associate degree	52	14.0	110.92 ± 15.42	1.167	0.313
Bachelor’s degree	315	84.7	114.70 ± 16.70		
Master’s degree	5	1.3	114.40 ± 16.35		
Professional title					
Primary Nurse	173	46.5	113.55 ± 17.53	1.482	0.229
Nurse-in-charge	152	40.9	113.68 ± 15.84		
Co-chief superintendent nurse or above	47	12.6	118.04 ± 14.68		
Length of service (years)					
≤ 5	76	20.4	114.11 ± 15.60	0.599	0.616
6–10	94	25.3	114.16 ± 17.29		
11–20	124	33.3	112.96 ± 17.58		
>20	78	21.0	116.17 ± 14.82		
Job duty					
Scrub nurse	121	32.5	116.10 ± 15.84	5.114	0.002
Circulating nurse	166	44.6	110.83 ± 16.42		
Equipment, consumables, and other auxiliary positions	32	8.6	114.72 ± 17.41		
Nursing manager	53	14.2	119.91 ± 16.06		
Marital status					
Unmarried	61	16.4	110.57 ± 15.85	1.895	0.152
Married	305	82.0	114.80 ± 16.67		
Others	6	1.6	118.67 ± 13.62		
Number of children					
0	83	22.3	111.24 ± 17.88	1.694	0.185
1	170	45.7	115.14 ± 15.44		
≥ 2	119	32.0	114.82 ± 16.99		
Monthly income (RMB Yuan)					
≤ 5,000	111	29.8	112.37 ± 17.16	5.069	0.007
5,001–10,000	211	56.7	113.52 ± 15.53		
>10,000	50	13.4	120.90 ± 17.91		
Average weekly resting days					
≤ 1	120	32.3	113.73 ± 16.08	0.062	0.940
1–2	109	29.3	114.34 ± 16.52		
≥ 2	143	38.4	114.41 ± 17.03		
Average daily working hours					
≤ 8	164	44.1	118.09 ± 15.35	8.579	<0.001
8–10	152	40.9	111.22 ± 17.11		
>10	56	15.1	110.71 ± 16.16		

### 3.2 Current situation of emotional labor strategies and QWL of OR nurses

The emotional labor strategies employed by OR nurses were primarily characterized by the expression of naturally felt emotions, with a mean score of 3.74 ± 0.69, followed by deep acting (3.36 ± 0.59) and surface acting (2.65 ± 0.62). The overall quality of work-life score was 114.17 ± 16.54. Among the dimensions of work-life quality, the average scores ranked from highest to lowest were as follows: job evaluation (3.62 ± 0.68), job control (3.62 ± 0.59), career satisfaction (3.59 ± 0.56), working conditions (3.58 ± 0.59), work-family balance (3.57 ± 0.77), and general happiness (3.49 ± 0.61). The mean score for work pressure was 2.80 ± 0.62.

### 3.3 Univariate analysis of QWL of OR nurses

The general demographic characteristics of the OR nurses were treated as independent variables, while the total QWL score was used as the dependent variable. The analysis revealed statistically significant differences in QWL scores based on job roles, monthly income, and average daily working hours (*p* < 0.05). Detailed results are presented in Table 1.

### 3.4 Correlation analysis between emotional labor strategies and QWL of OR nurses

The results of Pearson correlation analysis between different emotional labor strategies and work-life quality scores are presented in Table 2. Surface acting demonstrated a significant negative correlation with work-life quality (*r* = −0.437, *p* < 0.05), whereas deep acting and the expression of naturally felt emotions showed significant positive correlations with work-life quality (*r* = 0.291 and *r* = 0.457, respectively, *p* < 0.05).

**Table 2:** Correlation between emotional labor strategies and QWL of nurses in OR (r value).

	Surface acting	Deep acting	Expression of naturally felt emotions	QWL
Surface acting	1.000	–	–	–
Deep acting	0.011	1.000	–	–
Expression of naturally felt emotions	−0.468*	0.411*	1.000	–
QWL	−0.437*	0.291*	0.457*	1.000

*p < 0.05.

### 3.5 Analysis of factors affecting the quality of QWL of OR nurses

A multiple linear regression analysis was conducted with the total work-life quality score as the dependent variable. Independent variables included the general characteristics with statistically significant differences and the emotional labor strategies (surface acting, deep acting, and the expression of naturally felt emotions). The assignments of the independent variables are detailed in Table 3. The analysis results indicated that the factors included in the regression model were the expression of naturally felt emotions, surface acting, deep acting, average daily working hours, and monthly income (see Table 4).

**Table 3:** Assignment method of independent variables.

Independent variable	Assignment
Job duty	Scrub nurse =1, Circulating nurse =2, Equipment, consumables and other auxiliary positions =3, Nursing manager =4
Monthly income (RMB Yuan)	≦5,000 = 1, 5,001–10,000 = 2, >10,000 = 3
Average daily working hours (h)	≦8 = 1, 8–10 = 2, >10 = 3
Emotional labor strategy—surface acting	Original value
Emotional labor strategy—deep acting	Original value
Emotional labor strategy—expression of naturally felt emotions	Original value

**Table 4:** Multiple linear regression analysis of factors affecting QWL in OR nurses.

Independent variable	*B*	SE	*β*	*t*	*p*
Constant	95.633	7.105	–	13.461	<0.001
Score of expression of naturally felt emotions	1.861	0.437	0.232	4.259	<0.001
Score of surface acting	−1.091	0.192	−0.287	−5.676	<0.001
Score of deep acting	1.322	0.342	0.187	3.871	<0.001
Average daily working hours	−4.729	1.078	−0.204	−4.385	<0.001
Monthly income	4.472	1.204	0.173	3.714	<0.001

*F = 39.095, p < 0.001, R2 = 0.348, adjusted R2 = 0.339.*

## 4 Discussion

This study aimed to explore the complex relationship between emotional labor strategies and quality of QWL, as well as to identify the demographic and job-related factors that influence QWL. Emotional labor refers to the process by which workers manage their emotions to fulfill the emotional requirements of their roles, and it can be performed through various strategies, such as deep acting, surface acting, and the expression of naturally felt emotions. Furthermore, the study identified several significant predictors of QWL, including emotional labor strategies, job positions, monthly income, and average daily working hours. These demographic and job-related factors were found to influence how nurses perceive their work life quality.

In this survey, the QWL score for OR nurses was (114.17 ± 16.54), which was notably higher than the results reported by Zhu et al. (18). This discrepancy may be explained by regional differences, as well as variations in the demographic characteristics and professional contexts of the study participants. Specifically, factors such as healthcare infrastructure, organizational management practices, and cultural differences in work-life balance could contribute to these discrepancies in QWL scores across different regions. When comparing these results to findings from other studies, it is apparent that the QWL of nurses globally remains suboptimal, with a significant proportion of nursing staff reporting poor QWL (19–21). This global trend suggests that nurses across various healthcare settings are facing challenges in maintaining a satisfactory work-life balance. The persistent issue of low QWL among nurses is well-documented in the literature, reflecting widespread concerns regarding job stress, emotional strain, work overload, and insufficient organizational support. Such challenges underscore the need for comprehensive strategies to improve QWL for nurses, as their well-being is crucial not only for their own health but also for the delivery of high-quality patient care.

Upon analyzing the dimensions of QWL, we observed that the lowest scores were in the areas of work stress and overall happiness, with average scores of 2.80 ± 0.62 and 3.49 ± 0.61, respectively. OR nurses are required to have strong professional skills, be familiar with surgical procedures and surgeons’ preferences, and stay alert to manage emergencies during operations. Moreover, their workload is heavy, often requiring overtime. The demanding nature of their work leads to high stress levels and low happiness among OR nurses.

The factors influencing QWL are multifaceted and include organizational management quality, relationships with colleagues, job stability, occupational tasks, weekly workload, and personality traits (22–24). Single-factor analysis results revealed significant differences in the QWL of OR nurses based on job position, average daily working hours, and monthly income. Nurses in management positions reported higher QWL than their clinical counterparts, which aligns with findings by Misiak B (25). This could be because nurses in management roles mainly deal with administrative tasks, which typically involve a lighter clinical workload and lower emotional labor intensity compared to clinical nurses. As noted in previous research, heavy workload is a significant negative factor influencing QWL (26). Our study also showed that as the average daily working hours increased, QWL decreased. This finding corroborates the results from Bilal et al., which suggested that extra working hours negatively impact the work-life quality of healthcare workers (27). Higher income levels were generally associated with higher QWL, supporting the idea that appropriate compensation positively impacts the work-life quality of nurses (28). Our study suggests that nurses with higher monthly incomes reported better work-life quality, emphasizing the importance of fair and reasonable salary systems in improving QWL. Previous research by Wang et al. also demonstrated that salary directly influences nurses’ QWL (29). Therefore, nursing managers should ensure sufficient human resources in OR settings, minimize overtime hours, and implement fair performance evaluation systems to achieve equitable compensation, which in turn could improve nurses’ QWL.

This study results revealed that the most commonly used emotional labor strategy among OR nurses was the expression of naturally felt emotions, followed by deep acting and surface acting. These findings are consistent with those reported by Wang et al. (30). The expression of naturally felt emotions reflects a state where the nurse’s true feelings align with the emotional requirements of the organization (31). This emotional labor strategy suggests that nurses are in a relatively authentic and psychologically comfortable state at work. Deep acting was the second most frequently used emotional labor strategy, indicating that when confronted with emotional challenges, OR nurses adjust their internal emotions, accept the situation, and actively seek solutions. This approach is beneficial for the smooth progress of teamwork (32). On the other hand, surface acting involves suppressing one’s true emotions, and frequent use of this strategy is not conducive to improving QWL (33). Compared to surface acting, both deep acting and the expression of naturally felt emotions are considered more positive emotional labor strategies.

The results of this study emphasize the strong relationship between emotional labor strategies and QWL. Regression analysis identified emotional labor strategies as key predictors of QWL. Cheng et al. (34) argued that adopting appropriate emotional labor strategies contributes to better clinical nursing work, while Gao et al. (35) found that deep acting improves job satisfaction, while surface acting tends to reduce it. Given the close relationship between emotional labor strategies and QWL, it is crucial to explore measures to help nurses adopt effective emotional labor strategies. Enhancing organizational support may be one such measure. Several studies have shown that organizational support positively correlates with nurses’ emotional labor (36–40). For nursing managers, improving organizational support, providing emotional labor training, establishing effective incentive mechanisms, and helping nurses regulate their emotions are important steps toward improving QWL.

This study has several limitations that should be considered when interpreting the results. First, the cross-sectional design limits the ability to establish causal relationships between emotional labor strategies and quality of work life. Although regression analysis provided valuable insights into the association between these variables, the temporal sequence of these relationships cannot be determined. Future longitudinal studies would help to better understand the dynamic changes over time. Second, the sample was selected from a limited number of hospitals in a specific region, which may affect the generalizability of the findings. To enhance the external validity, future research should consider expanding the sample size and including participants from diverse geographical locations and healthcare settings. Third, this study relied on self-reported data, which may introduce response biases such as social desirability or recall bias. Incorporating objective measures and multi-source data in future studies could help to reduce these biases. Lastly, while the study explored a variety of demographic factors influencing QWL, other potentially relevant variables, such as organizational culture or job satisfaction, were not examined and could be important areas for future research.

## 5 Conclusion

In conclusion, this study highlights the significant relationship between emotional labor strategies and the quality of work life among operating room nurses. The findings demonstrate that emotional labor strategies, such as deep acting, surface acting, and the expression of naturally felt emotions, have a substantial impact on nurses’ QWL. Additionally, demographic factors such as job position, monthly income, and daily working hours were identified as key predictors of QWL. These results emphasize the need for healthcare organizations to recognize the importance of emotional labor in nursing practice and provide adequate support to enhance nurses’ work-life quality. Improving organizational support, offering training programs, and fostering a positive work environment can contribute to better emotional well-being and job satisfaction for nurses, ultimately improving patient care outcomes. Future research should explore longitudinal studies and expand the sample size to further understand the dynamic relationship between emotional labor strategies and QWL in diverse healthcare settings.

## Data Availability

The original contributions presented in the study are included in the article, further inquiries can be directed to the corresponding author.
